# Estrogen receptor α-NOTCH1 axis enhances basal stem-like cells and epithelial-mesenchymal transition phenotypes in prostate cancer

**DOI:** 10.1186/s12964-019-0367-x

**Published:** 2019-05-23

**Authors:** Yongmei Shen, Jiasong Cao, Zhixian Liang, Qimei Lin, Jianxi Wang, Xu Yang, Ran Zhang, Jiaojiao Zong, Xiaoling Du, Yanfei Peng, Ju Zhang, Jiandang Shi

**Affiliations:** 1 College of Life Sciences and Bioactive Materials Key Lab of the Ministry of Education, Nankai University, Tianjin, 300071 China; 2 National Engineering Research Center for Biomaterials, Sichuan University, Chengdu, 610064 China; 3 School of Integrative Medicine, Tianjin University of Traditional Chinese Medicine, Tianjin, 300193 China

**Keywords:** Prostate cancer, CD49f, EMT, Estrogen, ERα, NOTCH1

## Abstract

**Background:**

Prostate cancer (PCa) is the second leading cause of mortality and a leading cause of malignant tumors in males. Prostate cancer stem cells (PCSCs) are likely the responsible cell types for cancer initiation, clinical treatment failure, tumor relapse, and metastasis. Estrogen receptor alpha (ERα) is mainly expressed in the basal layer cells of the normal prostate gland and has key roles in coordinating stem cells to control prostate organ development. Here, we investigated the roles of the estrogen-ERα signaling pathway in regulating PCSCs.

**Methods:**

Correlation of CD49f and ERα/NOTCH1 was analyzed in human clinical datasets and tissue samples. Flow cytometry was used to sort CD49f^Hi^ and CD49f^Low^ cells. EZH2 recruitment by ERα and facilitation of ERα binding to the *NOTCH1* promoter was validated by Co-IP and ChIP. Primary tumor growth, tumor metastasis and sensitivity to 17β-estradiol (E2) inhibitor (tamoxifen) were evaluated in castrated mice.

**Results:**

ERα expression was significantly higher in CD49f^Hi^ prostate cancer basal stem-like cells (PCBSLCs), which showed basal and EMT features with susceptibility to E2 treatment. ERα-induced estrogen effects were suggested to drive the NOTCH1 signaling pathway activity via binding to the *NOTCH1* promoter. Moreover, EZH2 was recruited by ERα and acted as a cofactor to assist ERα-induced estrogen effects in regulating NOTCH1 in PCa. In vivo, E2 promoted tumor formation and metastasis, which were inhibited by tamoxifen.

**Conclusions:**

Our results implicated CD49f+/ERα + prostate cancer cells associated with basal stem-like and EMT features, named EMT-PCBSLCs, in heightened potential for promoting metastasis. NOTCH1 was regulated by E2 in CD49f^Hi^ EMT-PCBSLCs. These results contribute to insights into the metastatic mechanisms of EMT-PCBSLCs in PCa.

**Electronic supplementary material:**

The online version of this article (10.1186/s12964-019-0367-x) contains supplementary material, which is available to authorized users.

## Background

Prostate cancer (PCa) is the most commonly diagnosed cancer in aging males. Despite the advances in treatment, it remains the second leading cause of cancer-related death worldwide [1, 2]. Current PCa therapies include surgery, androgen blockade, radiation and chemotherapy. A suggested reason for the failure of these clinical therapies is the existence of cancer stem cells (CSCs), which lead to cancer recurrence and more aggressive progression [3, 4]. The origin of prostate cancer stem cells (PCSCs) remains controversial. PCSCs may arise from normal stem cells, which are situated within the basal layer of the prostate gland, and they share multiple properties with prostate basal layer cells [5, 6]. However, recent studies have also provided evidence that PCSCs may also originate from luminal cells. For example, PCSCs may arise from transformed epithelial cells through epithelial-mesenchymal transition (EMT) to acquire migratory and metastatic properties [7].

PCSCs possess cellular markers in common with normal stem cells, such as CD49f, CD44, CD117, CD133, TERT and p63 [8, 9]. CD49f is a basal stem-like cell marker, which plays important roles in cell-to-cell communication and cell motility [10, 11], and has been utilized as a target in the isolation of human prostate stem/progenitor cells [12, 13]. Evidence from a multitude of cancer studies showed that increased aggressiveness is accompanied by up-regulation of molecular trait characteristics common to stem cells. Recently, Smith et al reported that more aggressive PCa had higher proportions of CD49f^Hi^ basal stem cell subpopulations [14]. Several signaling pathways have been shown to play roles in promoting PCSCs generation, including androgen receptor (AR) [15], Pten [16], Wnt [17], Notch [18], and Hedgehog [19] related pathways, among others. However, the precise molecular mechanisms responsible for PCSCs generation have yet to be uncovered.

Prostate growth and development are controlled by the sex hormones, especially androgen and estrogen [20, 21]. The hormone receptors, which mediate the steroids’ effects, are differentially expressed in prostate epithelial cells. In the normal prostate, AR is predominantly expressed in luminal cells, estrogen receptor-α (ERα) expression is restricted to the basal cell layer which harbors prostate stem cells, and estrogen receptor-β (ERβ) is expressed in both basal layer cells and luminal cells [22, 23]. Both ERα and ERβ are reported to be expressed in PCa, and evidence indicates that higher-Gleason stage carcinomas had increased ERα expression and decreased ERβ expression [21]. It has also been reported that ERα expression was higher than that of AR expression in the basal cell subtypes, compared with luminal cells subtype within the prostate gland [24]. Moreover, the EMT process can endow cancer cells with stem cell properties [25] and our previous studies have shown that the estrogen-ERα signaling pathway enhanced EMT progression in benign prostate epithelial cells in vitro and in vivo [26, 27]. Thus, it can be speculated that ERα plays critical roles in enhancing the stemness of prostate cancer cells.

In this study, we demonstrated that CD49f+/ERα + prostate cancer cells associated with basal stem-like and EMT features (EMT-PCBSLCs), as having elevated potential for metastasis, NOTCH1 may be an essential E2-regulated gene pivotal to driving CD49f^Hi^ EMT-PCBSLCs subpopulations. The results shown here further insights into the molecular mechanisms of metastasis by EMT-PCBSLCs in PCa.

## Methods

### Cell culture and treatment

LNCaP-abl cells were generously gifted by Professor Helmut Klocker from the Innsbruck University School of Medicine. LNCaP-abl cells were cultured in RPMI-1640 medium (Sigma, Saint Louis, Missour, USA) supplemented with 100 mg/mL penicillin/streptomycin (P/S, HyClone, Logan, UT) and 10% charcoal dextran stripped fetal bovine serum (CDS FBS, Invitrogen, Carlsbad, CA). PC3 cells were purchased from the Deutsche Sammlung fuer Mikroorganismen and Zellkulturen (DSMZ, Braunschweig, Germany), LNCaP and 22RV1 cells were obtained from the American Type Culture Collection (ATCC, Manassas, VA) and were cultured in RPMI-1640 medium (Sigma) supplemented with 100 mg/mL P/S and 10% FBS (Invitrogen, Carlsbad, CA). Cells were routinely cultured in a CO_2_ incubator (5% CO_2_, 37 °C).

### Tumor sphere formation

LNCaP-abl cells were resuspended, dissociated into single cells and were cultured in 6-well ultra-low attachment culture plates (Corning, Amsterdam, The Netherlands) with DMEM/F-12 (Gibco, Carlsbad, CA) supplemented with 1% L-Glutamax, 1% P/S, 20 ng/μL human epidermal growth factor (hEGF; Gibco), 20 ng/μL basic fibroblasts growth factor (bFGF; Gibco), 1× B27 without vitamin A (Invitrogen), heparin sodium, 1× insulin-transferrin-selenium A (Invitrogen), 1× non-essential amino acids (Invitrogen), 0.1 mM β-mercaptoethanol (Sigma) and 10^3^ U/mL leukemia inhibitory factor (LIF; Millipore, Billerica, MA). Besides these, the concentration of E2 was 1 nM in the control group and 10 nM in the E2 group. Sphere cultures were seeded at a density of 2 × 10^3^ cells/mL and culture media was fully replaced every 96 h. Parental and prostasphere cultures were propagated for 12 days before the spheres were enzymatically dissociated using StemPro Accutase (Gibco) for second and third generation cell culture for 7 days, separately. Spheres were imaged and visualized via Olympus CX41 microscopy to evaluate the volume, or were collected for qRT-PCR or immunofluorescence detection.

### Histological and immunohistochemical (IHC) assay

Prostate tissue samples from CRPC patients (aged 63–78, *n* = 6) who underwent radical prostatectomy for prostate carcinoma (Gleason scores of 2–9, *n* = 14) were obtained from the Department of Pathology and the Department of Urology, the Second Affiliated Hospital of Tianjin Medical University (Tianjin, China). Normal human prostate specimens from six patients undergoing radical cystectomy for bladder cancer (aged 30–40, *n* = 6) were obtained from the Department of Urology, Shanghai First People’s Hospital (Shanghai, China). Men with chronic inflammation were excluded from this study. All samples were obtained with informed consent of patients and approval of the study was obtained from the Ethics Committee of Nankai University.

The use of tissue samples in this study was approved by the institutional review board. The primary prostate tumors were embedded in paraffin. Hematoxylin and eosin (HE) staining and IHC staining was performed as previously described [28]. The primary antibody dilutions used are provided in Additional file 5: Table S1. The stained slides were mounted and visualized under bright field an Olympus CX41 microscope.

### Immunofluorescence (IF) assay

LNCaP-abl or PC3 cells were cultured on slides and/or treated with DMSO (0.1% v/v) or 10 nM E2 for 72 h. Enriched stem cell spheres of LNCaP-abl (PCSCs) were attached to the bottom of confocal dishes, which were coated with polylysine for 30 min and with laminin for 4 h. IF assays were performed as previously described [28]. Images were taken using a fluorescence microscope (Leica, Germany) at an original magnification of 400×. Primary antibody dilutions are provided in Additional file 5: Table S1.

### Flow cytometry

Flow cytometry analysis was used to detect cell membrane expression of antigens, CD49f and/or NOTCH1, antibody staining was performed in HBSS/5% FBS or CDS FBS. After 15 min on ice, stained cells were washed to remove excess unbound antibodies before resuspension of cells in HBSS. Flow sorting used a BD FACSAriaII cell sorter (BD, San Jose, CA, USA), and analysis was performed using a FACSCalibur (BD).

For co-staining of CD49f and Vimentin, cells were disassociated and stained for CD49f before fixation in 4% formaldehyde followed by permeabilization in HBSS/0.2%TritonX-100. Next, cells were stained for Vimentin by incubation in antibody HBSS/5% FBS or CDS FBS solution for 30 min at room temperature. Cells were then washed with incubation buffer and incubated with the appropriate secondary fluorescent antibodies. Finally, the cells were washed and resuspended in HBSS and analysed on a FACSCalibur. The antibodies used are listed in Additional file 5: Table S1.

### siRNA and transient transfection

Control siRNA and siRNA specific to ERα were ordered from GenePharma (Shanghai, China). The sequences used to target ERα (siERα) were as follows: siERα 1#, 5′-UUCUCCGAACGUGUCACGUTT-3′; siERα 2#, 5′-GAUGAAAGGUGGGAUACGATT-3′. siEZH2 1#, 5′-GAAUGGAAACAGCGAAGGA-3′; siEZH2 2#, 5′-GACACCCGGUGGGACUCAGAAG-3′; Cells were seeded into 6-well plates at approximately 70–90% confluence and transfected with Lipofectamine 3000 (Invitrogen) and plasmid/siRNA at a ratio of 1:1. Transfected cells were harvested after 72 h of transfection for RNA or protein extraction. The concentration of plasmid was 2.5 μg for a 6-well plate, and the siRNA concentration was 75 pmol.

### Quantitative real-time polymerase chain reaction (qRT-PCR)

Total RNA was prepared from cells using TRIzol reagent (Invitrogen) and according to the manufacturer’s instructions. qRT-PCR was performed as described previously [29]. Following qRT-PCR analysis, the cycle threshold (Ct) was determined for the housekeeping gene, hypoxanthine phosphoribosyl transferase 1 (HPRT), as well as for target genes using auto-baseline and auto-threshold conditions (Primer Premier 5). Normalized gene expression data, obtained using ∆∆Ct (∆Ct reference-∆Ct target) and the formula 2^−∆∆Ct^, were utilized in subsequent analyses. Primer sequences are shown in Additional file 5: Table S2.

### Western blot analysis

Primary prostate tumor tissues (0.5 mg) were ground using a homogenizer in 500 μL of radioimmunoprecipitation assay buffer (RIPA, Thermo Fisher, Waltham, MA, USA), total protein was obtained, and Western blot analysis was performed as described previously [29]. The primary antibody dilutions used are provided in Additional file 1: Table S1.

### Chromatin immunoprecipitation (ChIP) and ChIP-re-ChIP assays

LNCaP-abl or PC3 cells were seeded in 100 mm dishes and treated with DMSO (0.1% v/v) or E2 for 30 min. EZH2 or ERα was knocked down using siRNA for 72 h, before harvest of LNCaP-abl or PC3 cells. ChIP experiments were performed following the Cold Spring Harbor (New York, USA) ChIP protocol with minor modifications [30]. ChIP-re-ChIP assays on supernatants were completed following a similar protocol to the primary ChIP experiments. Briefly, bead eluates from the first ChIP were incubated with 10 mM PMSF at 37 °C for 30 min and diluted 1:50 in dilution buffer (1% Triton X-100, 2 mM EDTA, 150 mM NaCl, 20 mM Tris-HCl at pH 8.1) followed by ChIP with the second antibodies [31]. Quantitative ChIP was performed using RT-PCR. The amounts of immunoprecipitated DNA were normalized to the input. The EZH2- or ERα-specific antibody and IgG utilized are shown in Additional file 5: Table S1. The NOTCH1 promoter primer is shown in Additional file 5: TableS2.

### Co-immunoprecipitation (Co-IP) assay

For Co-IP experiments, LNCaP-abl or PC3 cells were maintained in phenol red-free medium supplemented with 2.5% CDS FBS for 24 h and then treated with 10 nM E2 or DMSO (0.1% v/v) for 48 h. Cells were washed twice with ice-cold phosphate-buffered saline and then protein was extracted by lysis buffer (50 mM Tris, pH 8.0, 120 mM NaCl, 0.5% v/v Nonidet P-40, and protease inhibitor mixture) for 10 min on ice. Samples were then incubated with the anti-ERα and anti-EZH2 antibodies or control normal rabbit immunoglobulin G, overnight at 4 °C on a rotating platform, protein A beads were then added for 2 h at 4 °C. Beads were washed six times in washing buffer (20 mM Tris, pH 8.0, 1 mM EDTA, 900 mM NaCl, 0.5% v/v Nonidet P-40, and protease inhibitor mixture). Complexes were boiled in 5 × SDS-PAGE loading buffer for 5 min to elute target protein from the protein A beads. The immunoprecipitated samples were detected by Western blot.

### Bioinformatics analysis

Heat map analysis based upon mRNA-Seq cluster prostate adenocarcinoma (PRAD) samples of The Cancer Genome Atlas (TCGA) was divided into four clusters, and the indicated genes were selected to be analyzed. There were 498 PRAD samples. We also selected the top 10% of CD49f high- and low-expression from 498 PRAD samples individually, as based on the *z*-score analysis. For the correlation of CD49f, ERα and NOTCH1, we used mRNA expression data from human prostate cancer databases of the TCGA (num(Normal) = 52), num (PRAD) = 498)) and Genotype-Tissue Expression (GTEx, num (Normal) = 100)).

### Animal studies

The zs-green1 sequence was stably transferred and effectively expressed by lentiviral infection in LNCaP-abl cells, named LNCaP-abl-Green. 1 × 10^6^ LNCaP-abl-Green cells in 100% Matrigel (BD) were inoculated subcutaneously into the primary prostate of the castrated 6-week-old male Balb/c nude mice. One week later, E2 (48 μg/mL) was administered via silastic capsules (1.5 cm) implanted subcutaneously between the scapulae for 7 days, as previously described [32]. The mice were treated with tamoxifen (30 mg/kg, MCE, New Jersey, USA) every two days for 5 weeks. Images of different mice were captured and analyzed using an in vivo imaging system (Perkin Elmer IVISSPE). The tumor tissue was taken for extraction of total protein for Western blot or fixed with 4% paraformaldehyde, then embedded in paraffin for IHC or IF analyses. All animal experiments were approved by the Committee for Ethics in Animal Experimentation at the National Cancer Center.

### Statistical analysis

Data are shown as the means ± S.D. Statistical calculations were performed with GraphPad Prism 6 analytical tools. Significance was assessed using Student’s paired *t*-test, and the specific statistical test applied to the data was discribed in the figure legends. *, and *P* < 0.05 was considered statistically significant.

## Results

### CD49f^+^/ERα^+^ CSCs possess basal cell features

**Fig. 1:**
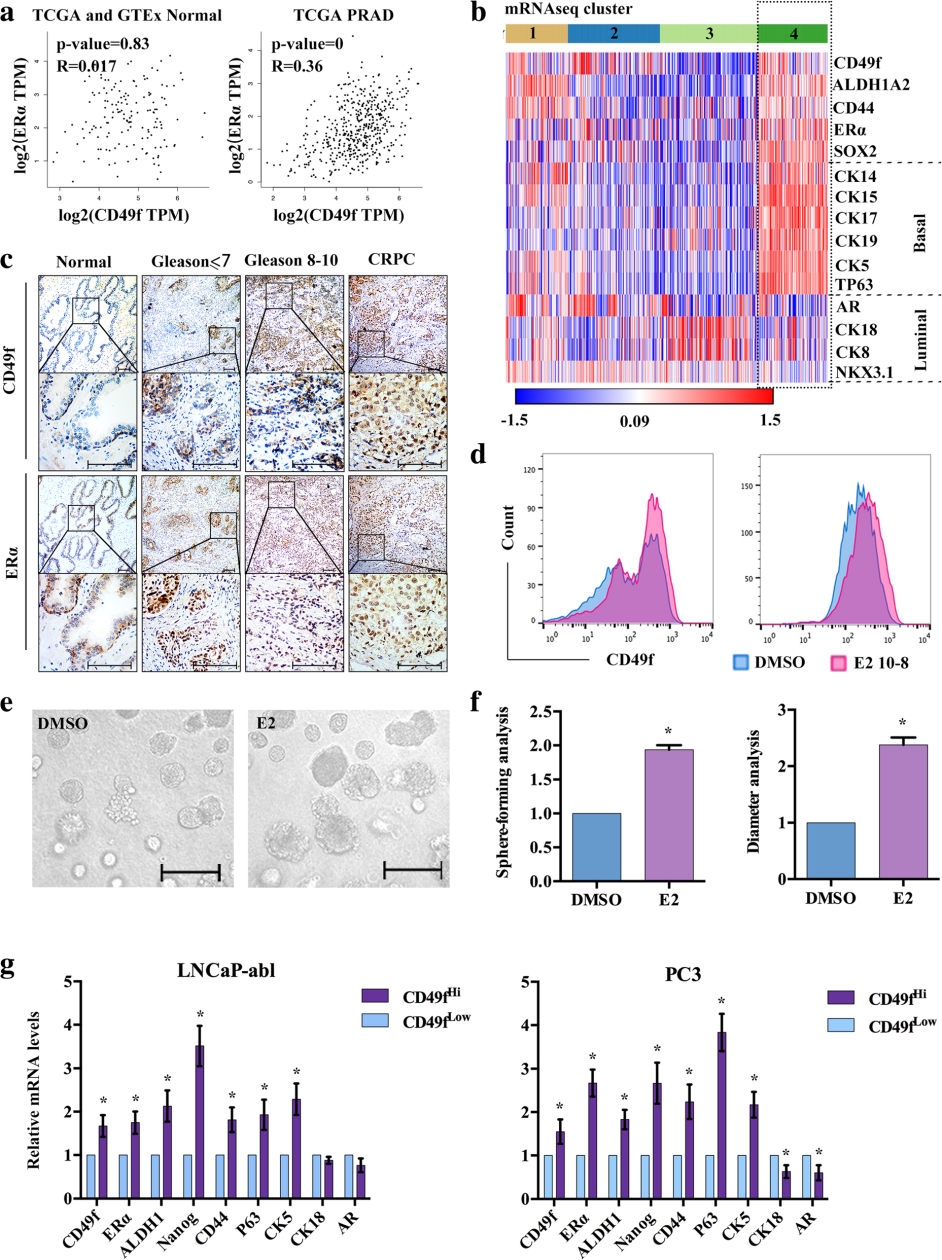
The expression of CD49f and ERα. **a**, The correlation of CD49f and ERα in normal prostate and PRAD patients from TCGA (num (Normal) = 52), num (PRAD) = 498)) and GTEx (num (Normal) = 100) datasets. **b**, Heat map analysis of the differentially expressed genes in PRAD patients. The TCGA consortium devised a subclassification of prostate cancers (num(N) = 497) into four distinct groups (1–4) based upon mRNA-Seq clustering and *z*-score analysis in Morpheus with a *P* ≤ 0.05 criterion, Rows: samples; columns: the indicated genes. **c**, IHC staining of CD49f and ERα in normal prostate tissues and cancer tissues with different Gleason score. Scale bar, 200 μm. **d**, Flow cytometry analysis of the expression of CD49f in LNCaP-abl and PC3 treated with either DMSO or 10 nM E2 for 72 h (*n* = 3). **e**, Analysis of enriching stem cell spheres treated with E2 for 2 weeks. Scale bar, 100 μm. **f**, Sphere-forming and diameter analysis of **e**. **g**, Flow cytometry sorting of CD49f^Hi^ and CD49f^Low^ cells in LNCaP-abl and PC3, qRT-PCR analysis showing expression changes of the indicated genes. The data are presented as the means±SD (*n* = 3). *, *P* < 0.05 vs. CD49f^Low^ PCBSLCs. Abbreviate: PRAD: Prostate Adenocarcinoma

We analyzed the correlation of CD49f and ERα from TCGA and GTEx, the results showed that the correlation of CD49f and ERα was higher in prostate cancer than that in normal prostate (Fig. 1a). The TCGA consortium devised a subclassification of prostate adenocarcinoma (PRAD) into four distinct groups (1–4) based upon mRNA-Seq cluster. Using the “1–4” designation, we clustered these patient samples with our PCa signature. Interestingly, as observed in Fig. 1b, tumors in cluster 4 appeared to have high-expression of *CD49f*, *ERα*, stem cell markers, basal markers and low-expression of luminal markers. We defined this group of patients as CD49f^+^/ERα^+^ expressing cells with stem and basal features (Fig. 1b). Next, we analyzed CD49f and ERα expression in a cohort of matched normal prostate tissue (*n* = 6), prostate adenocarcinoma (PCa) (*n* = 14) and CRPC (*n* = 6). CD49f and ERα were both significantly upregulated in prostate tumors compared with normal tissues. Furthermore, ERα expression was also positive at the region of CD49f^+^ expression, regardless of the different Gleason score of prostate tissues (Fig. 1c). We speculated that CD49f and ERα may have significant co-expression in prostate cancer. The IF results showed that CD49f and ERα could be co-expressed in prostate tissues and enriched in LNCaP-abl stem cells (Additional file 1: Figure S1A).

In the TCGA prostate dataset, mean expression of the *ERα* gene was significantly higher in the top 10% of CD49f high expression tumors than in the top 10% of CD49f low expression tumors (Additional file 1: Figure S1B). The expression of *ERα* was higher in androgen independence than androgen dependent PCa cell lines (Additional file 1: Figure S1C). To investigate whether exogenous estrogens play a role in prostate cancer, we used flow cytometry to detect the expression of CD49f in androgen independent PCa cell lines, LNCaP-abl and PC3, the results showed that CD49f-positive cells were significantly increased after treatment with E2 (Fig. 1d), and the results of enriched stem cell spheres of LNCaP-abl (PCSCs) treated with E2 showed that both the number and diameter of stem cell spheres was increased following treatment with E2 (Fig. 1e, f). The heat maps indicated that the expression of stem cell and basal markers were higher, and luminal markers were lower in the top 10% of CD49f^Hi^ than in the top 10% of CD49f^Low^ samples (Additional file 1: Figure S1D). Then, we sorted CD49f^Hi^ and CD49f^Low^ cells from LNCaP-abl and PC3 cells and observed that the expression of *CD49f*, *ERα*, stem cell and basal markers in CD49f^Hi^ cells were higher than in CD49f^Low^ cells, whereas luminal markers showed a contrasting trend (Fig. 1g, Additional file 1: Figure S1E). These results showed that tumors and cells with CD49f^+^/ERα^+^ were expressive for basal stem-like features and were susceptible to E2.

### ERα-induced estrogen effects enhance EMT in CD49f^Hi^ PCBSLCs

**Fig. 2:**
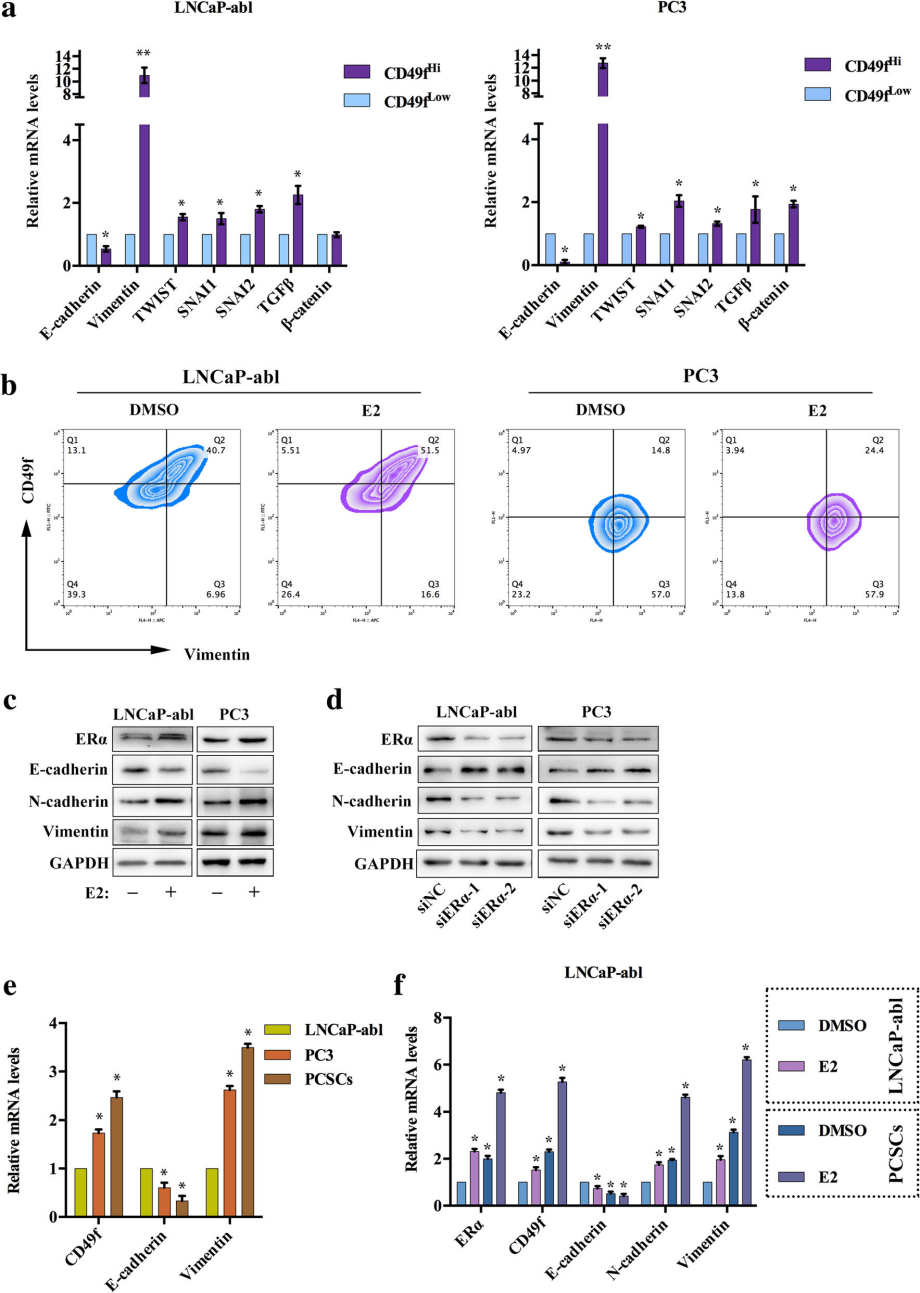
E2 promotes EMT in CD49f^Hi^ PCBSLCs. **a**, qRT-PCR analysis showing expression changes of the indicated genes in the sorted CD49f^Hi^ and CD49f^Low^ PCBSLCs. The data are presented as the mean ± SD (*n* = 3). *, *P* < 0.05 vs. CD49f^Low^ PCBSLCs. **b**, Flow cytometry analysis of the coexpression of CD49f and Vimentin in LNCaP-abl and PC3 cells treated with either DMSO or 10 nM E2 for 72 h (*n* = 3). **c** and **d**, Western blot analysis the indicated proteins in (**c**) LNCaP-abl or PC3 treated with DMSO or 10 nM E2 treatments for 72 h; (**d**) LNCaP-abl or PC3 with ERα knockdown (*n* = 3). **e** and **f**, qRT-PCR analysis showing expression changes of EMT markers in LNCaP-abl and enriched stem cell spheres of LNCaP-abl (PCSCs) (**e**), and treated with 10 nM E2 or DMSO (**f**). The data are presented as the means±SD (*n* = 3). *, *P* < 0.05 vs. DMSO

We examined the expression of EMT markers in the sorted cells and observed that the expression of *E-cadherin* was decreased, whereas *Vimentin*, *TWIST*, *SNAI1*, *SNAI2*, *TGFβ* and *β-catenin* were increased in CD49f^Hi^ PCBSLCs, compared to CD49f^Low^ PCBSLCs (Fig. 2a). Vimentin is a well-known mesenchymal marker that is often used as an EMT marker. Therefore, we used flow cytometry to detect the co-expression of CD49f and Vimentin, the results showed that the numbers of CD49f and Vimentin double-positive cells were increased after treatment with E2 (Fig. 2b). Thus, we hypothesized that estrogen promoted EMT in PCa. Western blot analysis confirmed that E2 could decrease the expression of E-cadherin, a hallmark of the EMT process, while the expression levels of N-cadherin and Vimentin were increased (Fig. 2c). The expression of E-cadherin was up-regulated, and N-cadherin and Vimentin was down-regulated in LNCaP-abl and PC3 cells, following ERα knockdown (Fig. 2d). We compared LNCaP-abl cells and enriched stem spheres of LNCaP-abl, and the results showed that the expression of *N-cadherin* and *Vimentin* in PC3 cells were higher than in LNCaP-abl cells, and were highest in PCSCs. As expected, the expression of *E-cadherin* was lower in PC3 cells than that in LNCaP-abl cells, and was lowest in PCSCs (Fig. 2e). Furthermore, the EMT induction by E2 was more evident in PCSCs than LNCaP-abl cells (Fig. 2f). In addition, the expression changes of the stem cell, EMT, basal and mature luminal markers induced by E2 could be reduced following NOTCH1 knockdown in LNCaP-abl cells (Fig. 2g). Both of the TCGA consortium of PRAD clusters and the top 10% of CD49f high- and low-expressing cells showed that the expression markers of metastases and EMT were higher in cluster 4 and CD49f^Hi^ samples (Additional file 2: Figure S2A, B). These results indicated that the ERα-induced estrogen effect enhanced EMT in CD49f^Hi^ PCBSLCs.

### NOTCH1 is closely associated with CD49f and responds to E2 in PCa

**Fig. 3:**
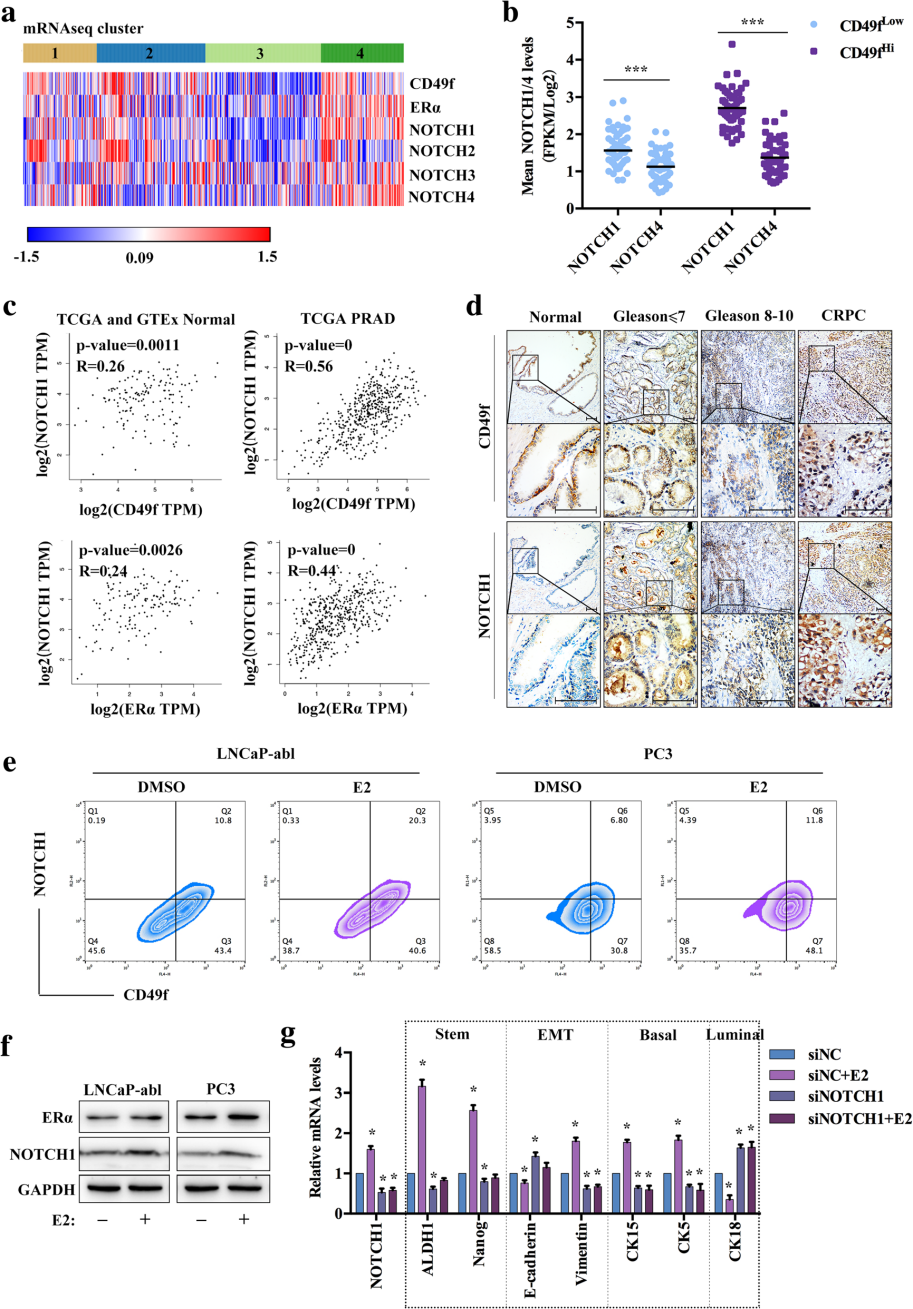
The expression of CD49f and NOTCH1. **a**, Heat map analysis of the differentially expressed genes in PRAD patients from TCGA. **b**, Mean mRNA analysis of *NOTCH1* and *NOTCH4* in PRAD of TCGA. Significance was assessed using Student’s paired *t*-test. The scatter dot plot is presented as the median. ***, *P* < 0.001. **c**, The correlation of CD49f or ERα and NOTCH1 in normal prostate and PRAD from TCGA (num (Normal) = 52), num (PRAD) = 498)) and GTEx (num (Normal) = 100)). **d**, IHC staining of CD49f and NOTCH1 in normal prostate tissues and cancer tissues with different Gleason score. Scale bar, 200 μm. **e**, Flow cytometry analysis of the coexpression of CD49f and NOTCH1 in LNCaP-abl and PC3 treated with either DMSO or 10 nM E2 for 72 h (*n* = 3). **f**, Western blot analysis of the indicated proteins in LNCaP-abl or PC3 cells treated with 10 nM E2 treatments for 72 h (*n* = 3). **g**, qRT-PCR analysis showing expression changes of the indicated genes in LNCaP-abl treated with either DMSO or 10 nM E2 for 72 h. The data are presented as the means±SD (*n* = 3). *, *P* < 0.05 vs. siNC

The TCGA consortium results showed that both of the expression of NOTCH1 and NOTCH4 was highest and most significant in the cluster 4 (Fig. 3a). In the TCGA prostate dataset, the mean expression levels of the *NOTCH1* and *NOTCH4* genes were significantly higher within the top 10% of CD49f high expression tumors than the top 10% of CD49f low-expression tumors. The expression of *NOTCH1* was higher than *NOTCH4* in both CD49f high and low-expression samples (Fig. 3b), which was also confirmed in LNCaP-abl, PC3, and stem cells enriched from LNCaP-abl cells (Additional file 3: Figure S3A). Thus, we decidedly focused on the relationship between NOTCH1 and CD49f/ERα. We analyzed the correlation of CD49f/ERα and NOTCH1 from TCGA, and the results showed that the correlation of CD49f/ERα and NOTCH1 was more significant in PRAD than that in normal healthy prostate tissue (Fig. 3c). The expression of CD49f and NOTCH1 in human PCa tissues (*n* = 14) was higher than in healthy prostate tissue (*n* = 6), whilst their expression was increased with the Gleason score in PCa (*n* = 14), and was the highest in CRPC (*n* = 6). Furthermore, NOTCH1 expression was also detected at the region of CD49f-positive expression, regardless of different Gleason scores of the prostate tissues (Fig. 3d). The IF analyses supported the finding that CD49f and NOTCH1 were co-expressed in the human PCa tissues, as well as PCSCs (Additional file 3: Figure S3B). This provided us with the speculation that NOTCH1 may be regulated by estrogen in PCa. Therefore, we used flow cytometry to detect the co-expression of CD49f and NOTCH1 protein. Results showed that the number of CD49f and NOTCH1 double-positive cells was increased in LNCaP-abl and PC3 cells, when treated with E2 (Fig. 3e). We detected the expression of NOTCH1 treated with E2 in LNCaP-abl and PC3 cells in order to explore whether E2 regulated NOTCH1. The results showed that E2 promoted the expression of NOTCH1 (Fig. 3f). In LNCaP-abl cells treated with E2, the expression of *NOTCH1*, *Vimentin*, stem cell and basal markers were up-regulated, whereas the expression of *E-cadherin* and luminal markers were down-regulated. Interestingly, the changes in gene expression induced by E2 were inhibited by *NOTCH1* knockdown in LNCaP-abl cells (Fig. 3g). These results illustrated that NOTCH1 was closely associated with CD49f in PCa and responded to E2 treatment.

### EZH2 acts as a cofactor to assist ERα-induced estrogen effects regulating NOTCH1 in PCa

**Fig. 4:**
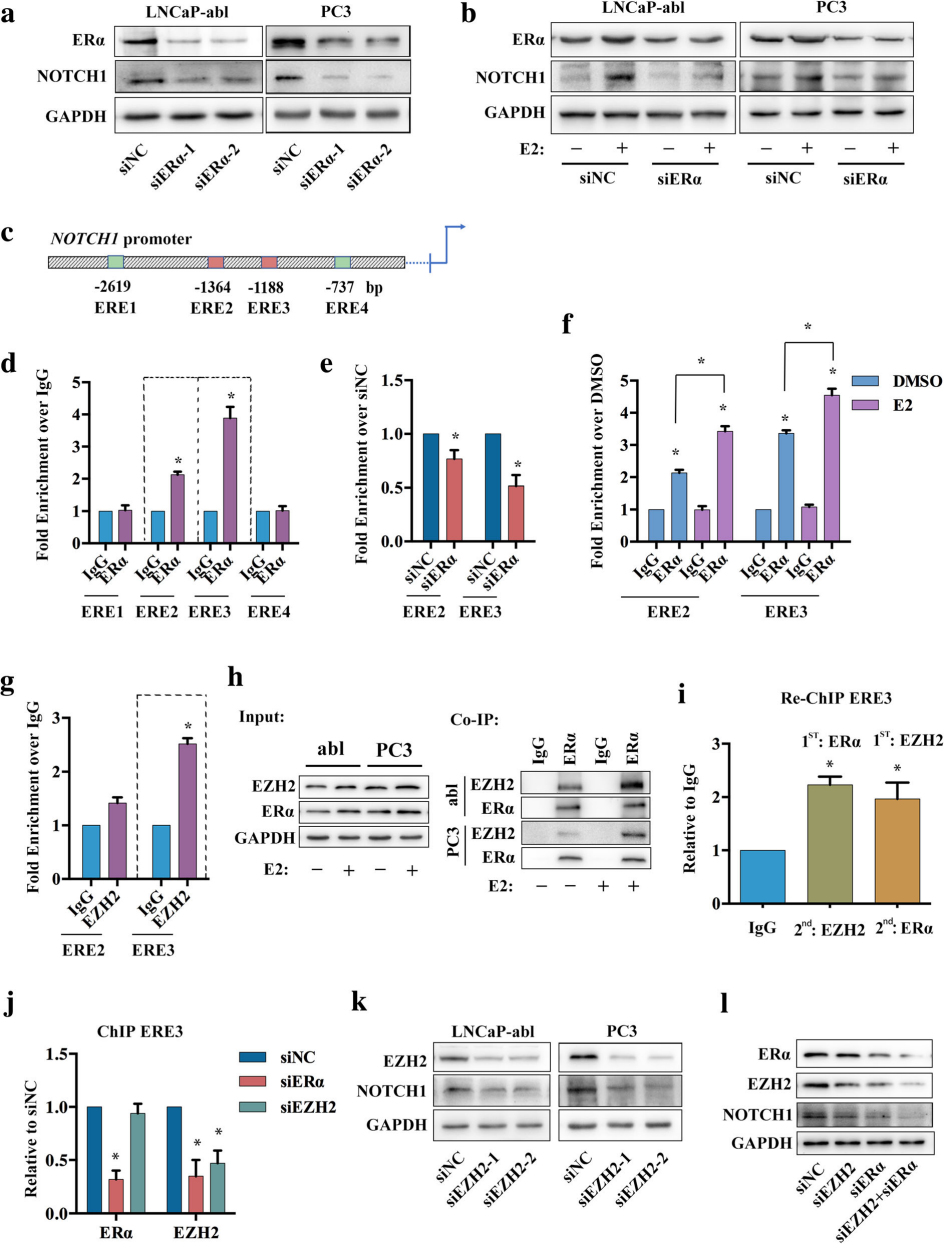
The mechanism of E2 regulated NOTCH1. **a**, Western blot analysis of the indicated proteins in LNCaP-abl or PC3 cells with ERα knockdown (*n* = 3). **b**, Western blot analysis of the indicated proteins in LNCaP-abl or PC3 with or without ERα knockdown after treatment with either DMSO or 10 nM E2 for 72 h (*n* = 3). **c**, Diagram of the *EZH2* promoter regions analyzed for EREs in the ChIP assays. **d**-**f**, Anti-ERα antibodies were used for ChIP assays regarding the EREs (ERE1-ERE4) of the *NOTCH1* promoter in LNCaP-abl (**d**), LNCaP-abl with ERα knockdown (**e**), and LNCaP-abl treated with DMSO or E2 for 30 min before being harvested (**f**). qRT-PCR amplification was performed using a series of primers targeting EREs. The data are presented as the mean ± SD (*n* = 3). *, *P* < 0.05 vs. IgG (**d**), siNC (**e**), or DMSO (**f**). **g**, Anti-EZH2 antibodies were used for ChIP assays regarding the ERE2 and ERE3 of the *NOTCH1* promoter in LNCaP-abl. The data are presented as the mean ± SD (*n* = 3). *, P < 0.05 vs. IgG. **h**, Co-IP analysis of the interaction between ERα and EZH2 in LNCaP-abl or PC3 cells following treatment with either DMSO or 10 nM E2 for 72 h (*n* = 3). Abbreviation: abl: LNCaP-abl. **i** and **j**, ChIP-re-ChIP qPCR analysis of ERE3 of *NOTCH1* promoter in LNCaP-abl (**i**) and ChIP qPCR analysis of LNCaP-abl with ERα or EZH2 knockdown (**j**). The data are plotted as the mean ± SD (*n* = 3). *P < 0.05 vs. IgG (**i**) and siNC (**j**). **k** and **l**, Western blot analysis of the indicated proteins in LNCaP-abl with ERα or/and EZH2 knockdown (*n* = 3)

To explore the molecular mechanism of E2 regulation of NOTCH1, ERα was knocked down in LNCaP-abl and PC3 cells. The results showed that expression of NOTCH1 was down-regulated in LNCaP-abl and PC3 cells with ERα knockdown (Fig. 4a). Next, we knocked down ERα in LNCaP-abl and PC3 cells treated with or without E2. E2 promoted NOTCH1 expression, whereas ERα downregulation reduced NOTCH1 expression. ERα knockdown notably reduced the effects of E2 on LNCaP-abl and PC3 cells (Fig. 4b). We assessed the *NOTCH1* promoter for the presence of estrogen-response elements (EREs), which facilitate the direct binding of ERα to the promoter sequence. Analysis revealed the presence of four putative EREs (Fig. 4c). The results of ChIP assays on LNCaP-abl cells showed that there was enrichment for endogenous ERα protein bound to the *NOTCH1* promoter at ERE2 and ERE3 (− 1364 and − 1188 bp, respectively) (Fig. 4d). Furthermore, ChIP PCR for ERE2 and ERE3 enrichment by ERα was decreased when ERα was knocked down and increased when LNCaP-abl cells were treated with E2 (Fig. 4e, f).

EZH2 has been reported to bind to the *NOTCH1* promoter in TN breast cancer [33], and to bind to ERα protein [34]. ChIP analyses showed that EZH2 could bind to the *NOTCH1* promoter at the ERE2 (− 1188 bp) site, in LNCaP-abl cells (Fig. 4g). Co-IP analysis showed that E2 promoted ERα binding to EZH2, in LNCaP-abl and PC3 cells (Fig. 4h). Therefore, we hypothesized that ERα and EZH2 formed a complex that facilitated binding to the *NOTCH1* promoter, and the ChIP-re-ChIP results confirmed our hypothesis (Fig. 4i). Subsequently, we knocked down ERα or EZH2 expression to determine which protein was predominantly responsible for the ERα-EZH2 complex binding to the *NOTCH1* promoter. No change in ERα binding to the *NOTCH1* promoter was observed when EZH2 was knocked down, whereas EZH2 exhibited decreased binding to the *NOTCH1* promoter when ERα was knocked down (Fig. 4j). However, the expression of NOTCH1 was decreased with the knockdown of EZH2 and the most significant decrease was observed when both ERα and EZH2 were knocked down (Fig. 4k, l), suggesting that both ERα and EZH2 are required to activate *NOTCH1* transcription. These results indicated that ERα-induced estrogen effects may promote the NOTCH1 signaling pathway via binding and activation of the *NOTCH1* promoter. Furthermore, EZH2 is recruited by ERα and acts as the co-factor to assist ERα-induced estrogen effects in regulating NOTCH1 in PCa.

### Tamoxifen inhibits the growth and metastasis of prostate tumors

**Fig. 5:**
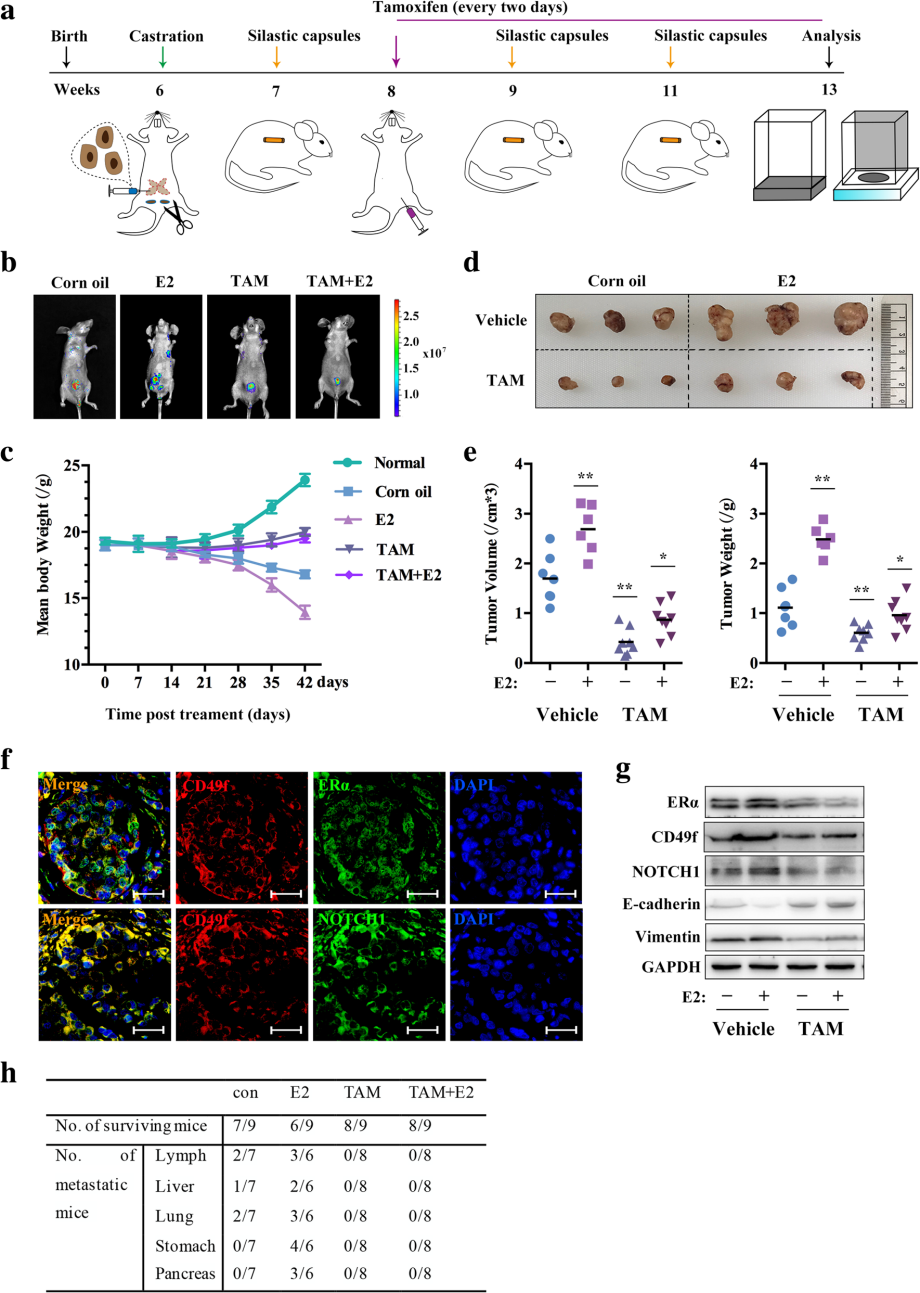
Tamoxifen inhibits the growth and metastasis of prostate tumors in vivo. **a**, Schematic illustration of the experimental strategy. **b**, LNCaP-abl-Green cells were implanted into nude mice, with 9 castrated mice in each group. Tumor growth and metastasis were detected with an IVIS system. The data are presented as the mean ± SD. **c**, Changes in body weight over time (*n* = 3). **d** and **e**, Three primary tumors were selected randomly to be photographed with a digital camera (**d**), and the volume and weight of the primary tumors were measured (**e**). The scatter dot plot is presented as the median. *P < 0.05; **P < 0.01 vs. Vehicle+PBS. **f**, IF analysis of CD49f and ERα or NOTCH1 co-expression in prostate primary tumors tissues of the E2 group. **g**, Western blot analysis of the indicated proteins in prostate primary tumors tissues (*n* = 3). **h**, Number of surviving and metastatic mice injected with LNCaP-abl-Green cells in different groups. Abbreviation: TAM: tamoxifen

For in vivo analyses, we injected LNCaP-abl-Green cells into the prostates of castrated nude mice, and after one week, E2 was administered via silastic capsules implanted subcutaneously between the scapulae for every 2 weeks. Corn oil was used for the control treatment group. The corn oil and E2 groups were randomly divided into 2 sub-groups and treated with either tamoxifen or 1 × PBS every two days for 5 weeks. Images of the different mice at the end of the experiment were captured and analyzed using an in vivo imaging system. The mice were weighed weekly for the duration of the experiment, as shown in the schematic diagram (Fig. 5a). The in vivo bioluminescence imaging results demonstrated that the tumors of mice treated with estrogen showed significant increases in epic-fluorescent intensity of the primary and metastasized points, which were significantly reduced in the tamoxifen group (Fig. 5b). We also observed weight loss of mice was most notable in the E2 treatment group. In contrast, the weight of mice in the tamoxifen group did not notably change (Fig. 5c). The tumors were explanted, measured, and the results of the tumor weight and volume analysis showed that E2 promoted tumor growth, which was inhibited by tamoxifen (Fig. 5d, e).

We assessed the co-expression of CD49f and other genes in primary tumors of mice. The results showed that CD49f, ERα and NOTCH1 were significantly co-expressed in the primary tumor tissues (Fig. 5f). IHC analysis of the primary tumor tissues showed that estrogen promoted the expression of CD49f, ERα, NOTCH1, and Vimentin, and that the expression of E-cadherin was decreased; the changes induced by E2 were significantly inhibited by tamoxifen (Additional file 4: Figure S4), and the Western blot results showed the same trends (Fig. 5g). Furthermore, statistical analysis of metastatic tissues showed that estrogen promoted metastasis to the lymph nodes, liver and lung tissues as well as in the stomach and pancreas in some cases. Metastases to the lymph nodes and the liver were the most severe. In contrast, there were no or few metastases in the tamoxifen-treated groups, regardless of whether E2 was added or not (Fig. 5h). These results demonstrated that tamoxifen inhibited the formation and metastasis of prostatic neoplasms induced by E2.

## Discussion

Besides for the abnormal activation of androgen signaling pathway through mechanisms such as mutation in AR, dysregulation of AR, AR cofactors and so on, the existence of CSCs may also cause the failure of anti-androgen therapy [35, 36]. PCSCs share multiple properties with prostate basal stem cell populations, ERα has been reported to be higher and AR to be lower in the basal cell subtypes [24]. We found that the basal stem cell marker, CD49f, had a positive correlation with ERα expression, and the TCGA consortium results showed that patients in cluster 4 had a high-expression of CD49f, ERα, stem cell markers, basal markers, and low-expression of luminal markers included AR. We also observed that the expression of ERα in CD49f^Hi^ PCBSLCs was higher than in CD49f^Low^ PCBSLCs and AR was lower in CD49f^Hi^ PCBSLCs on the contrary. Moreover, E2 could increase CD49f-positive cells and affected the formation of stem cell spheres. Therefore, we primarily investigated how ERα-induced estrogen effect played a role in driving CD49f^Hi^ PCBSLCs sub-populations in the present study.

Only CSCs- but not the non-CSCs- within the tumor have unlimited potential to be able to seed new tumors, and therefore, targeting CSCs capacity to drive tumor growth and metastasis has received much attention from the research community [37]. CSCs and EMT often accompany each other in the promotion of metastasis [7, 25, 38]. Our results showed that CD49f-positive PCBSLCs were a cell subpopulation, with a high degree of EMT potential, and that ERα-mediated estrogen effects strongly promoted an EMT response in CD49f^Hi^ PCBSLCs. Although CD49f is found in a much larger cell population than just stem cells, high CD49f expression has a predictive value for biochemical recurrence and disease-specific death [39]. High CD49f expression has been declared to enhance invasion and tumor-initiating cell activities in metastatic breast cancer [40]. The results reported here illustrated that the estrogen signaling pathway apart from AR, Pten, Wnt, Notch and so on had clear relevance in CD49f^Hi^ EMT-PCBSLCs and the progression of PCa metastasis.

There are increasing data showing the Notch signaling pathway’s essential functionality in normal, healthy prostate development [41]; and that its activity was increased and implicated in advanced metastatic disease [42] and putative PCSCs [43]. Expression changes associated with stem cells, EMT, basal and luminal cell types when induced by E2, and these changes were also shown to be prevented by *NOTCH1* mRNA knockdown, which suggested that NOTCH1 was regulated by E2 in CD49f^Hi^ EMT-PCBSLCs with ERα high-expression. It has been reported that Notch signaling governs commitment of the mouse mammary stem cells-enriched subpopulation to the luminal cell lineage, which included Notch1 involvement [44]. Notch1 intracellular domain (N1ICD) could rescue the capacity of putative prostate luminal progenitors for unipotent differentiation [45]. *Notch1* gene expression was also reported to be evident within the basal cell compartment and overexpressed in a CD49f^Hi^ basal stem cell population [14, 42]. The TCGA consortium results showed that tumors in cluster 1 and 2 also appeared to have elevated expression of *NOTCH1* compared with cluster 3, but lower than cluster 4 (Fig. 3a). Cluster 4 was the tumor type with basal features, but also EMT and stem-like characteristic marker expression, thus we choose this cluster to investigate in the present study.

It has been reported that E2 induced the recruitment of N1ICD to the *Hes-1* promoter in breast cancer cells [46], and that Notch1 regulated expression levels of ERα in ER-positive breast cancer [47]. However, research on the regulation of ERα-induced estrogen effects on Notch1 have received little attention in the prostate and PCa studies. We found that E2 regulated NOTCH1 via ERα binding to the *NOTCH1* promoter. ERα expression levels were higher in the basal layer of prostate cancer, whilst expression was observed in the luminal layer of breast cancer [24], suggesting that the mechanisms of E2 regulating Notch1 in PCa may be tissue type dependent, a point that warrants further investigation. In addition, EZH2 was recruited by ERα and acted as a co-factor to assist ERα-induced estrogen effects in the regulation of NOTCH1, suggesting a new approach for the treatment of PCa. RNA Polymerase II with EZH2 binding was previously reported to be important for binding and activation of the *NOTCH1* promoter in breast CSCs [48] which supports our conclusions to a certain extent. These results showed that the NOTCH1 signaling pathway may be regulated predominantly by E2 in EMT-PCBSLCs.

AR plays a central role in the progression of PCa. It has been reported that the mutant AR could be activated by other steroids, even anti-androgens [35]. Here the estrodial’s treatment and/or ERα knockdown experiments were performed in both AR-positive LNCaP-abl cells and AR-negative PC3 cells. The results suggested that estrogen enhanced CSCs and EMT processes primarily mediated through ERα, but not AR in vitro. In the in vivo experiments, we found that E2 promoted the formation and metastasis of tumors. Following tamoxifen treatment, we observed poorly differentiated areas and clear infarctions in the primary tumors, and tamoxifen effectively inhibited E2-induced tumor growth and metastasis. Tamoxifen is one of the most widely used drugs in the treatment of ER-positive breast cancer, and mechanisms for acquired resistance to tamoxifen during treatment are largely unknown. However, it has been suggested that Notch1 was partly responsible for tamoxifen resistance [47]. Although the location of ERα protein expression was different in prostate cancer and breast cancer [24], tamoxifen could inhibit the expression of NOTCH1 in PCa, in this study. Accumulating evidence indicates that EMT and CSCs play important roles during the development of metastasis of PCa; the inhibition of EMT and CD49f by tamoxifen could explain metastasis induced by E2, which in-turn was also inhibited by tamoxifen treatment. Results of in vivo experiments further confirmed that ERα-induced estrogen effect played an important part in PCa, beyond AR.

## Conclusions

**Fig. 6:**
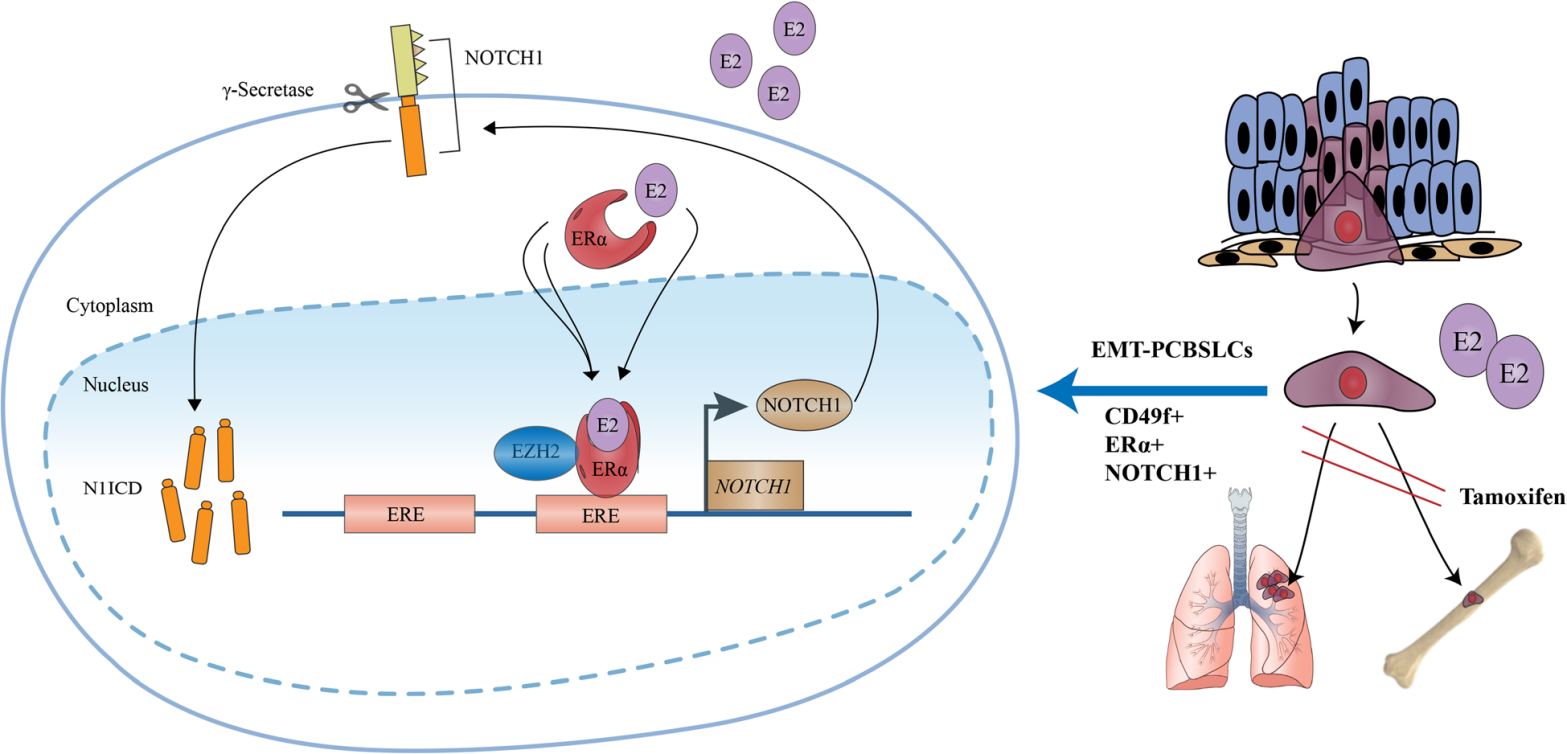
The total signaling pathway. ERα-induced estrogen effects promoted the expression of NOTCH1 by binding *NOTCH1* promoter at − 1364 and − 1188 bp, EZH2 was recruited by ERα and acts as a cofactor to assist ERα-induced estrogen effects in regulating NOTCH1 in PCa. The ERα-induced estrogen effects may promote CD49f^Hi^ EMT-PCBSLCs metastasis to other organs

Our results identify CD49f^Hi^ EMT-PCBSLCs as a cell sub-type with high ERα expression, EMT and metastasis features. ERα-induced estrogen effects drove the expression of NOTCH1 via the binding and transcriptional activation of the *NOTCH1* promoter. Moreover, EZH2 was recruited by ERα and acted as a co-factor to assist ERα-induced estrogen effects in regulating NOTCH1 in PCa. In vivo, E2 promoted tumor formation and metastasis, which was inhibited by tamoxifen in a NOTCH1-dependent manner (Fig. 6). The findings of this investigation, that the estrogen signaling pathway is involved in the promotion of EMT-PCBSLCs sub-populations with ERα high-expression, implicate and reveal new targets for therapeutic intervention for PCa metastasis.

## Additional files

Additional file 1: **Figure S1. A**, IF analysis of CD49f and ERα co-expression in prostate tissues, enriched stem cell spheres of LNCaP-abl (PCSCs), LNCaP-abl or PC3 cells. Scale bar, 50 μm. **B**, Mean mRNA analysis of *CD49f* and *ERα* in PRAD of TCGA. Significance was assessed using Student’s paired *t*-test. The scatter dot plot is presented as the median. ***, *P* < 0.001. **C**, qRT-PCR analysis showing expression changes of *ERα* in the PCa cell lines and PCSCs. The data are presented as the mean ± SD (*n* = 3). *, *P* < 0.05 vs. LNCaP. A-de: Androgen dependence; A-inde: Androgen independence. **D**, Heat map analysis of the differentially expressed genes in the top10% of CD49f high- and low-expression individually from 498 PRAD samples in TCGA. Based on the *z*-score analysis in Morpheus with a *P* ≤ 0.05 criterion, Rows: samples; columns: the indicated genes; Red, top 10% of CD49f high-expression; green, top 10% of CD49f low-expression. **E**, Flow cytometry sorted CD49f^Hi^ PCBSLCs of LNCaP-abl and PC3 cells (*n* = 3). Abbreviate: A-de: androgen-dependent; A-inde: androgen-independent. (TIF 3400 kb)

Additional file 2: **Figure S2. A**, Heat map analysis of the differentially expressed genes in PRAD patients. The TCGA consortium devised a subclassification of prostate cancers (num(N) = 497) into four distinct groups (1–4) based upon mRNA-Seq clustering and *z*-score analysis in Morpheus with a *P* ≤ 0.05 criterion, Rows: samples; columns: the indicated genes. **B**, Heat map analysis of the differentially expressed genes in the top 10% of CD49f high- and low-expression individually from 498 PRAD samples in TCGA. Based on the *z*-score analysis in Morpheus with a *P* ≤ 0.05 criterion, Rows: samples; columns: the indicated genes; Red, top 10% of CD49f high-expression; green, top10% of CD49f low-expression. (TIF 6540 kb)

Additional file 3: **Figure S3. A**, qRT-PCR analysis showing expression changes of *NOTCH1* and *NOTCH4* in LNCaP-abl, PC3 and PCSCs. The data are presented as the mean ± SD (*n* = 3). *, *P* < 0.05 vs. NOTCH1 expression of LNCaP-abl. **B**, IF analysis of CD49f and NOTCH1 co-expression in prostate tissues and PCSCs. Scale bar, 50 μm. (TIF 2100 kb)

Additional file 4: **Figure S4.** IHC staining showing the indicated antigens in prostate primary cancer tissues. Scale bar, 200 μm. (TIF 8560 kb)

Additional file 5: **Table S1.** List of antibodies. **Table S2.** Primer sequences. (DOC 73 kb)

## Abbreviations

abl: LNCaP-abl
AR: Androgen receptor
ChIP: Chromatin Immunoprecipitation
Co-IP: Coimmunoprecipitation
CSCs: Cancer stem cells
E2: 17β-Estradiol
EMT: Epithelial-mesenchymal transition
EREs: Estrogen-response elements
ERα: Estrogen receptor α
IF: Immunofluorescence
IHC: Immunohistochemical
N1ICD: NOTCH1 intracellular domain
PCa: Prostate cancer
PCBSLCs: Prostate cancer basal stem-like cell
PCSCs: Prostate cancer stem cells (enriched stem cell spheres of LNCaP-abl)
RT-PCR: Quantitative real-time reverse-transcription-polymerase chain reaction
TAM: Tamoxifen
TCGA: The Cancer Genome Atlas
