# Supplementary figures and images for: Estrogen receptor α-NOTCH1 axis enhances basal stem-like cells and epithelial-mesenchymal transition phenotypes in prostate cancer
Source: Cell Commun Signal. 2019 May 23;17:50. doi: 10.1186/s12964-019-0367-x (PMC6533681; doi:10.1186/s12964-019-0367-x)

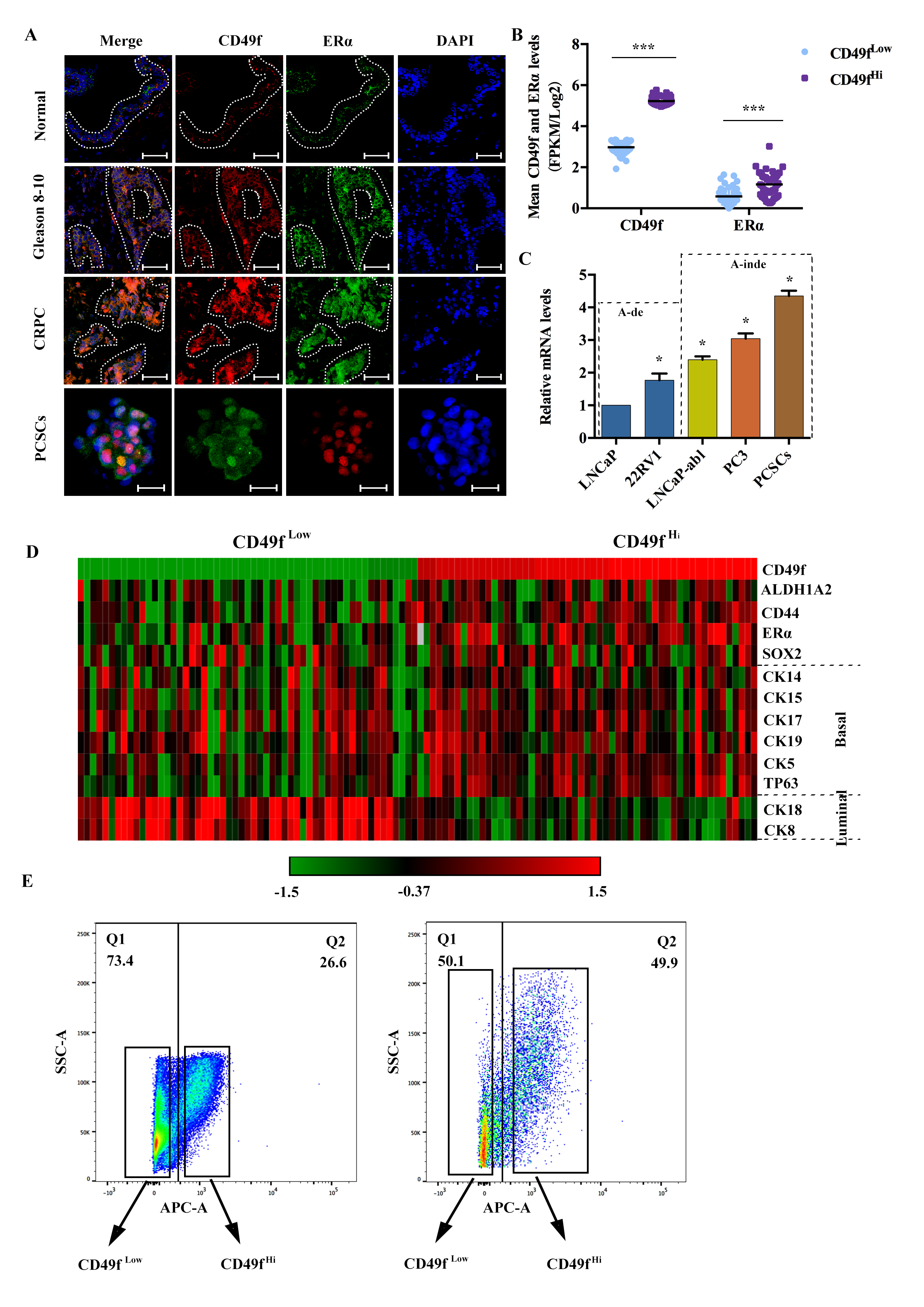

Supplement: Supplementary file 1 — Figure S1. A, IF analysis of CD49f and ERα co-expression in prostate tissues, enriched stem cell spheres of LNCaP-abl (PCSCs), LNCaP-abl or PC3 cells. Scale bar, 50 μm. B, Mean mRNA analysis of CD49f and ERα in PRAD of TCGA. Significance was assessed using Student’s paired t-test. The scatter dot plot is presented as the median. ***, P < 0.001. C, qRT-PCR analysis showing expression changes of ERα in the PCa cell lines and PCSCs. The data are presented as the mean ± SD (n = 3). *, P < 0.05 vs. LNCaP. A-de: Androgen dependence; A-inde: Androgen independence. D, Heat map analysis of the differentially expressed genes in the top10% of CD49f high- and low-expression individually from 498 PRAD samples in TCGA. Based on the z-score analysis in Morpheus with a P ≤ 0.05 criterion, Rows: samples; columns: the indicated genes; Red, top 10% of CD49f high-expression; green, top 10% of CD49f low-expression. E, Flow cytometry sorted CD49fHi PCBSLCs of LNCaP-abl and PC3 cells (n = 3). Abbreviate: A-de: androgen-dependent; A-inde: androgen-independent. (TIF 3400 kb) [file 12964_2019_367_MOESM1_ESM.tif]

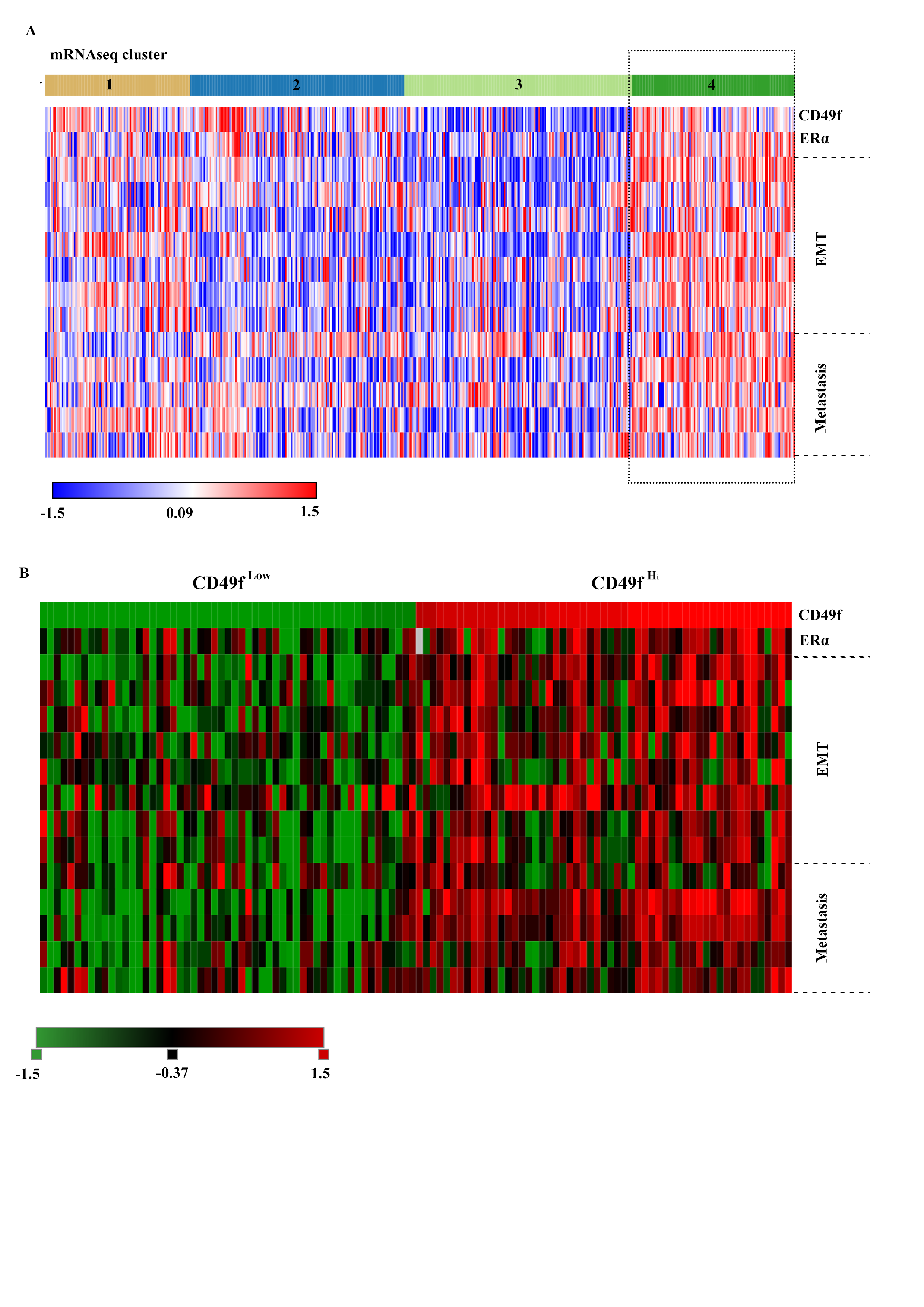

Supplement: Supplementary file 2 — Figure S2. A, Heat map analysis of the differentially expressed genes in PRAD patients. The TCGA consortium devised a subclassification of prostate cancers (num(N) = 497) into four distinct groups (1–4) based upon mRNA-Seq clustering and z-score analysis in Morpheus with a P ≤ 0.05 criterion, Rows: samples; columns: the indicated genes. B, Heat map analysis of the differentially expressed genes in the top 10% of CD49f high- and low-expression individually from 498 PRAD samples in TCGA. Based on the z-score analysis in Morpheus with a P ≤ 0.05 criterion, Rows: samples; columns: the indicated genes; Red, top 10% of CD49f high-expression; green, top10% of CD49f low-expression. (TIF 6540 kb) [file 12964_2019_367_MOESM2_ESM.tif]

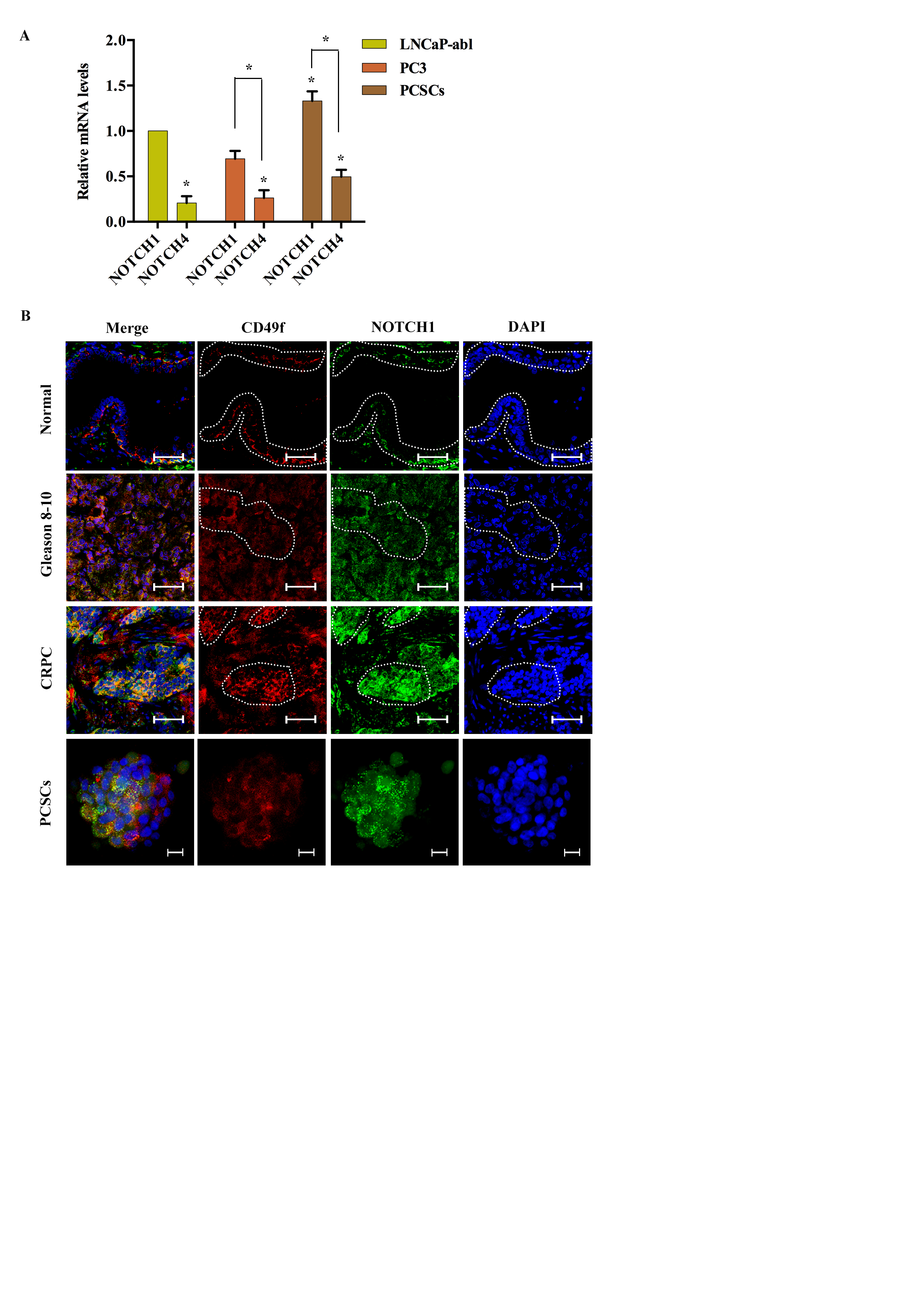

Supplement: Supplementary file 3 — Figure S3. A, qRT-PCR analysis showing expression changes of NOTCH1 and NOTCH4 in LNCaP-abl, PC3 and PCSCs. The data are presented as the mean ± SD (n = 3). *, P < 0.05 vs. NOTCH1 expression of LNCaP-abl. B, IF analysis of CD49f and NOTCH1 co-expression in prostate tissues and PCSCs. Scale bar, 50 μm. (TIF 2100 kb) [file 12964_2019_367_MOESM3_ESM.tif]

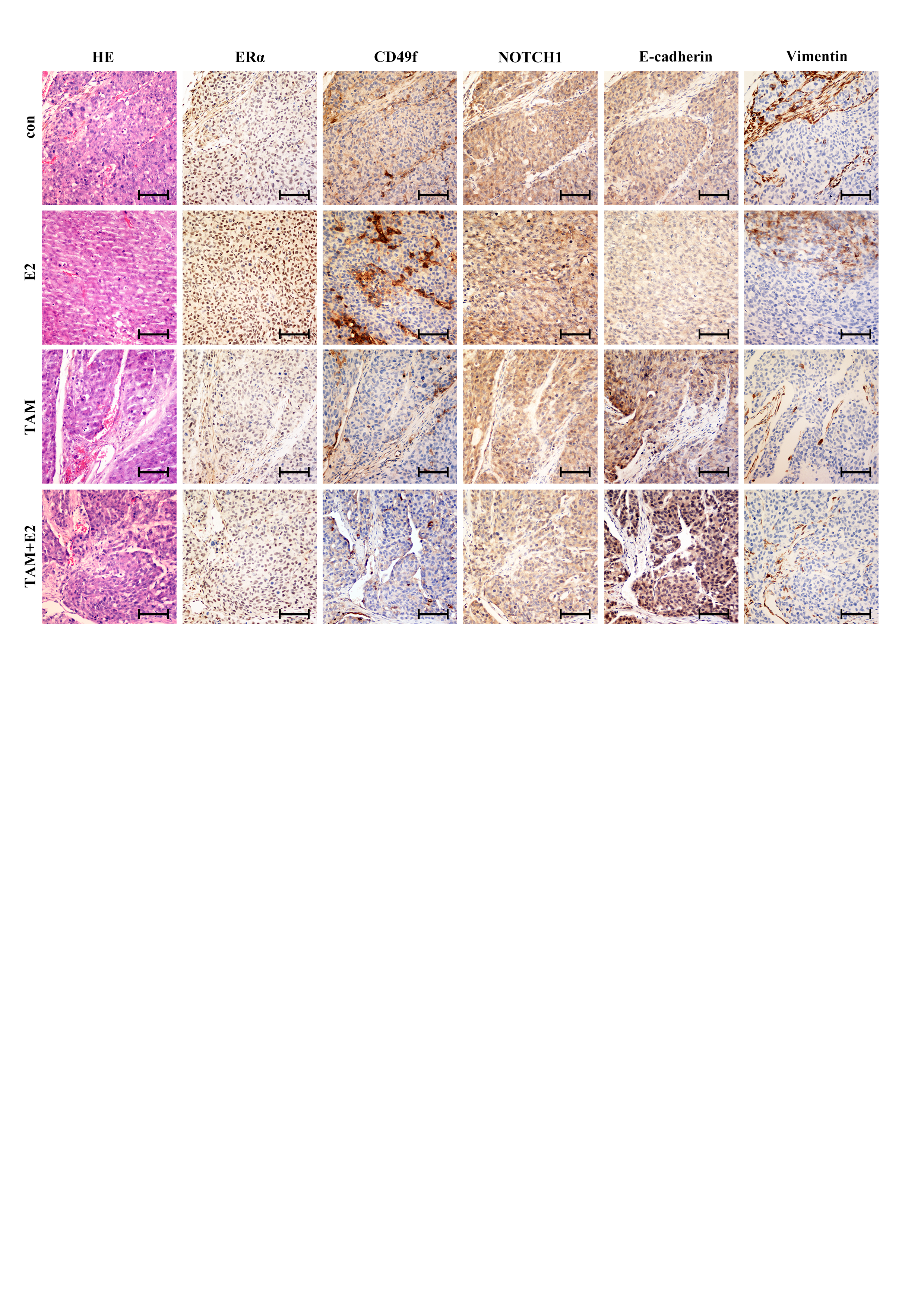

Supplement: Supplementary file 4 — Figure S4. IHC staining showing the indicated antigens in prostate primary cancer tissues. Scale bar, 200 μm. (TIF 8560 kb) [file 12964_2019_367_MOESM4_ESM.tif]
