# Supplementary material for: Estrogen receptor α-NOTCH1 axis enhances basal stem-like cells and epithelial-mesenchymal transition phenotypes in prostate cancer
Source: Cell Commun Signal. 2019 May 23;17:50. doi: 10.1186/s12964-019-0367-x (PMC6533681; doi:10.1186/s12964-019-0367-x)
Supplement: Supplementary file 5 — Table S1. List of antibodies. Table S2. Primer sequences. (DOC 73 kb) [file 12964_2019_367_MOESM5_ESM.doc]

**Supplementary Tables**

**Supplementary Table 1: List of antibodies**

| Peptide/Protein Target | Manufacturer, Catalog Number | Used |
| --- | --- | --- |
| CD49f | Biolegend, 313616  Abcam, ab194969  Abcam, ab181551 | APC, Flow Cytometry  Mouse, IHC/WB/IF  Rabbit, IF |
| ERα | Abcam, ab32063  Santa, sc-542 | Rabbit, IHC/WB/ChIP/CO-IP/IF  Mouse, IF |
| GAPDH | KANG CHEN, KC-5G4 | Mouse, WB |
| E-cadherin | BD, 610181 | Mouse, WB |
| N-cadherin | BD, 610920 | Mouse, WB |
| Vimentin | CST, 5741S  Proteintech, 60330-1 | Rabbit, WB/ Flow Cytometry  Mouse, IF |
| Notch1 | Abcam, ab8925 | Rabbit, IHC/WB/IF/Flow Cytometry |
| EZH2 | CST, 5246S | Rabbit, WB/ChIP |

Abbreviation: WB: Western Blot; IHC: Immunohistochemical; IF: Immunofluorescence.

**Supplementary Table 2: Primer sequences**

|  | sense | | antisense | purpose | |
| --- | --- | --- | --- | --- | --- |
| HPRT | | TGACACTGGCAAAACAATGCA | GGTCCTTTTCACCAGCAAGCT | qRT-PCR |  |
| CD49f | | ATGCACGCGGATCGAGTTT | TTCCTGCTTCGTATTAACATGCT | qRT-PCR |  |
| ERα | | GGACCATATCCACCGAGTCCTG | GCCTCCCCCGTGATGTAATAC | qRT-PCR |  |
| ALDH1 | | CTTGAATCCCGAATGGAAAGGG | GTGTATATCCCAGGGTGATCCTC | qRT-PCR |  |
| Nanog | | TTTGTGGGCCTGAAGAAAACT | AGGGCTGTCCTGAATAAGCAG | qRT-PCR |  |
| CD44 | | TCCAACACCTCCCAGTATG | TTCTGGACATAGCGGGTG | qRT-PCR |  |
| P63 | | GGACCAGCAGATTCAGAACGG | AGGACACGTCGAAACTGTGC | qRT-PCR |  |
| CK5 | | ACAATGTCAAGAAACAGTGCG | TGACTGGTCCAACTCCTTCTC | qRT-PCR |  |
| CK15 | | CTGGTAGTGTTTTCGGTGG | TTGAGCCTGAAGTCGTCC | qRT-PCR |  |
| CK18 | | GTATGAGGCCCTGCTGAA | CACCACTTTGCCATCCAC | qRT-PCR |  |
| AR | | TGGCGGCATGGTGAGCAGAG | AGGCAGGTCTTCTGGGGTGGAAA | qRT-PCR |  |
| E-cadherin | | GCCTTATGATTCTCTGCTCGTGT | TTGCCCCATTCGTTCAAGTAGT | qRT-PCR |  |
| Vimentin | | GGATGTTTCCAAGCCTGAC | AAGGGCATCCACTTCACA | qRT-PCR |  |
| Twist | | GGAGTCCGCAGTCTTACGAG | TCTGGAGGACCTGGTAGAGG | qRT-PCR |  |
| snail1 | | CCCAGTGCCTCGACCACTAT | GCTGGAAGGTAAACTCTGGATTAGA | qRT-PCR |  |
| slug | | CGAACTGGACACACATACAGTG | CTGAGGATCTCTGGTTGTGGT | qRT-PCR |  |
| TGFβ | | TGGACATCAACGGGTTCACT | GCAGAAGTTGGCATGGTAGC | qRT-PCR |  |
| β-catenin | | GAAACGGCTTTCAGTTGAGC | CTGGCCATATCC ACCAGAGT | qRT-PCR |  |
| Notch1 | | AAGCTGCATCCAGAGGCAAAC | TGGCATACACACTCCGAGAACAC | qRT-PCR |  |
| Notch4 | | TGCGAGGAAGATACGGAGTG | GGACGGAGTAAGGCAAGGAG | qRT-PCR |  |
| EZH2 | | CAGCCTTGTGACAGTTCGT | AGATGGTGCCAGCAATAGA | qRT-PCR |  |
| ERE1 | | CTCCTTAGCTCACCCTGACAATG | TGCACCCCAGGAGACTGAACT | ChIP |  |
| ERE2 | | GCCTGGCTGCTGTTACATAAAC | CTGGGGTAACATCTTGGGGTC | ChIP |  |
| ERE3 | | ACTGGCTGTTTCCAGAGTGC | GGCACAGAAAGCAGGACAAA | ChIP |  |
| ERE4 | | CCGACCCGTTTGTGCTTTCTG | TGCCTCGCGGACGGATTGT | ChIP |  |
